# Extensive lipoma-like changes of myxoid liposarcoma: morphologic, immunohistochemical, and molecular cytogenetic analyses

**DOI:** 10.1007/s00428-015-1721-z

**Published:** 2015-02-04

**Authors:** Hiroshi Iwasaki, Masako Ishiguro, Jun Nishio, Mikiko Aoki, Ryohei Yokoyama, Koichiro Yokoyama, Kenichi Taguchi, Kazuki Nabeshima

**Affiliations:** 1 Department of Pathology, Fukuoka University School of Medicine, 7-45-1 Nanakuma, Jonan-ku, Fukuoka 814-0180 Japan; 2 Department of Orthopedic Surgery, Fukuoka University School of Medicine, 7-45-1 Nanakuma, Jonan-ku, Fukuoka 814-0180 Japan; 3 Department of Orthopedic Surgery, National Hospital Organization Kyushu Cancer Center, 3-1-1 Notame, Minami-ku, Fukuoka 811-1395 Japan; 4 Department of Pathology, National Hospital Organization Kyushu Cancer Center, 3-1-1 Notame, Minami-ku, Fukuoka 811-1395 Japan

**Keywords:** Myxoid liposarcoma, Dedifferentiated liposarcoma, Lipoma-like liposarcoma, *FUS-DDIT3*, Fusion gene

## Abstract

Myxoid liposarcomas (MLSs) with extensive lipoma-like changes (MLSLC) are rare, and it is often difficult to distinguish them from well-differentiated liposarcoma (LS)/dedifferentiated LS (WDLS/DDLS) with myxoid changes. For the characterization of these neoplasms, we studied 8 MLSLCs, 11 ordinary MLSs, 4 WDLSs, and 6 DDLSs. Cytogenetically, MLSLC and ordinary MLS were characterized by t(12;16)(q13;p11) and *FUS-DDIT3* fusion gene, whereas WDLS/DDLS lacked the fusion gene but possessed giant marker/ring chromosomes. Both lipoma-like and myxoid components of the same MLSLC exhibited the identical *FUS-DDIT3*, as confirmed by fluorescence in situ hybridization (FISH) and reverse transcription polymerase chain reaction (RT-PCR). Immunohistochemically, MDM2 and CDK4 were positive in WDLS/DDLS but negative in MLSLC and ordinary MLS. PPARγ, C/EBPα, adipophilin, and perilipin were found in each type of LS. Adipophilin was expressed chiefly in tiny fat droplets of immature lipoblasts, whereas perilipin showed a strong positive staining in large fat vacuoles of signet ring and multivacuolated lipoblasts. The Ki-67 labeling index was lower in the lipoma-like component of MLSLC when compared with the myxoid component of the same tumors as well as ordinary MLS (*p* < 0.001). When compared with ordinary MLS, MLSLC may be less aggressive in clinical behavior (rare recurrences or metastases) after a wide surgical excision. In conclusion, the distinction between MLSLC and WDLS/DDLS is important, because of the differences of molecular cytogenetic features as well as clinical behaviors between these distinct sarcomas presenting similar morphologic features. In addition, the combined immunohistochemical detection of adipophilin and perilipin may provide a useful ancillary tool for identification of lipoblastic cells in soft tissue sarcomas.

## Introduction

Myxoid liposarcoma (MLS) is the second most common type of liposarcoma (LS) after atypical lipomatous tumor/well-differentiated LS (ALT/WDLS) [1, 2]. MLS usually presents as a large painless mass within the deep soft tissue of the extremities in young adults and middle-aged individuals. Histologically, MLS is composed of predominantly uniform round- to oval-shaped primitive mesenchymal cells admixed with signet ring or multivacuolated lipoblasts in a prominent myxoid stroma, which is rich in a delicate arborizing “chicken-wire” capillary vasculature. Included in this category are lesions formerly known as round cell LS (RCLS) characterized by hypercellular round cell morphology, which is associated with a significantly poor prognosis [1–3].

Although a variable number of signet ring lipoblasts are found in MLS, extensive mature adipocytic differentiation forming a lipoma-like component is very rare [4–6]. Morphologically, it is often difficult to distinguish MLS with extensive lipoma-like changes (MLSLC) from ALT/WDLS with myxoid changes, but the severe degree of nuclear atypia may help to exclude MLS/MLSLC [7]. Cytogenetically, most cases of MLS show specific chromosomal translocation t(12;16)(q13;p11) resulting in the formation of a *FUS-DDIT3* (also known as *TLS-CHOP*) fusion transcript [8–17], although occasional tumors exhibit the variant t(12;22) (q13;q12), producing an *EWSR1-DDIT3* fusion transcript [1, 18]. On the other hand, ALT/WDLS is characterized by supernumerary ring chromosome and/or giant markers with amplification of the 12q13 ~ q15 region, which is often associated with overexpression of *CDK4* and *MDM2* [1, 17, 19–23].

Myxoid areas within ALT/WDLS and dedifferentiated LS (DDLS) sometimes possess a prominent plexiform vascularity with thin-walled arborizing capillaries, creating a resemblance to MLS especially when interspersed small fat cells are present [7, 24–26]. On the basis of molecular and immunohistochemical studies, de Vreeze et al. [24] suggested that apparent primary retroperitoneal MLS/RCLS could be recognized as WDLS/DDLS with morphological features mimicking MLS/RCLS. They considered that finding of the MLS-specific translocations (fusion genes) in a retroperitoneal LS is highly suggestive of metastasis and should prompt search for a primary lesion outside the retroperitoneum.

In addition, rare cases of mixed-type LS composed of an admixture of MLS and WDLS have been reported by some investigators [17, 27]. More recently, Deyrup et al. [28] reported a new group of “fibrosarcoma-like lipomatous neoplasm,” which were composed of low-grade spindle cells showing varying degrees of lipoblastic differentiation and sometimes accompanied by abundant myxoid stoma and thin-walled arborizing capillaries similar to those of MLS, but these tumors lacked molecular cytogenetic characteristics of other types of lipomatous tumors.

The development of normal fat cells is considered to be regulated by various factors including peroxisome proliferator-activated receptor-γ (PPARγ) and CCAAT/enhancer-binding protein-α (C/EBPα) [29]. PPARγ may regulate the expression of lipid droplet-associated proteins including adipophilin and perilipin in normal tissues [30, 31], but little is known about the mechanism of adipocytic differentiation in MLSLC.

In order to clarify the true nature of rare lipomatous differentiation in MLS, we studied eight cases of MLSLC, by using immunohistochemistry (MDM2, CDK4, PPARγ, C/EBPα, adipophilin, perilipin, and Ki-67), chromosome analysis, fluorescence in situ hybridization (FISH), and reverse transcription polymerase chain reaction (RT-PCR). In addition, ordinary MLS (11 cases), WDLS (4 cases), and DDLS (6 cases) were studied as controls. To our knowledge, this is the first report describing the detailed molecular cytogenetic and clinicopathologic features of MLSLC.

## Materials and methods

### Tumor material and patient data

The consultation and archival files of molecular cytogenetic analysis of soft tissue tumors in the Department of Pathology, Fukuoka University School of Medicine, between 1987 and 2012 were searched for MLS with or without well-differentiated lipoma-like components. Eight cases of MLSLC were selected for the present study. In addition, 11 cases of ordinary MLS without lipoma-like components, 4 cases of WDLS, and 6 cases of dedifferentiated LS (DDLS) with myxoid changes were studied as controls.

Clinical parameters, including gender, age, location, and macroscopic features, were obtained from medical records. In all cases, histologic sections were available for pathologic review, and the diagnosis was confirmed according to the WHO (2013) classification. Formalin-fixed and paraffin-embedded tumor tissues available in each case were used for immunostaining and molecular analysis. In 16 cases, fresh tumor tissues were utilized for chromosomal analysis as well as molecular study. Follow-up information was obtained from the referring clinicians and from the existing medical records in accord with institutional guidelines.

### Immunohistochemical staining

For immunohistochemistry, 3-μm-thick paraffin-embedded tissue sections were mounted on silane-coated glass slides, deparaffinized, and heated in antigen retrieval buffer using a pressure cooker for 10 min or a microwave for 30 min. The following primary antibodies were used: MDM2 (dilution 1/100; Calbiochem, Darmstadt, Germany), CDK4 (dilution 1/200; Invitrogen, Camarillo, CA), PPARγ (dilution 1/100; Perseus Proteomics, Tokyo, Japan), C/EBPα (dilution 1/200; Cell Signaling Technology, Danvers, MA), adipophilin (dilution 1/20; Fitzgerald, Acton, MA), perilipin (dilution 1/200; Cell Signaling Technology, Danvers, MA), and Ki-67 (clone MIB-1, dilution 1/200; Dako, Glostrup, Denmark). Immunohistochemical staining was performed by using the Nichirei Histofine Simple Stain MAX PO (MULTI) (Nichirei Biosciences Inc., Tokyo, Japan). The reactions were visualized with diaminobenzidine, and the sections were counterstained with Mayer’s hematoxylin. For MDM2, CDK4, PPARγ, C/EBPα, adipophilin, and perilipin, the immunoreactivity was graded semiquantitatively as negative (0 %), 1+ (<25 % of neoplastic cells reactive), 2+ (25 to 50 % of neoplastic cells reactive), and 3+ (>50 % of neoplastic cells reactive). The Ki-67 labeling index was obtained as a percentage of positive nuclei by counting 500 neoplastic cells within the areas exhibiting the highest labeling index (hot spots). The Mann-Whitney *U* test was used to compare the differences of the Ki-67 index.

### Cytogenetic analysis

Primary cell cultures, harvesting, and preparation of slides were carried out as previously described [9, 21, 22]. Chromosome analysis was performed on GTG-banded (Giemsa/trypsin) metaphases, and the karyotypes were described according to the International System for Human Cytogenetic Nomenclature [32].

### FISH analysis for *DDIT3* break apart

FISH was performed on paraffin-embedded tissue sections (cases 1–8, 12, 13, 17, 18, 21–25), by using a commercially available *DDIT3* (*CHOP*) (12q13) Dual Color, Break Apart Rearrangement Probe (Abbott Molecular Inc.). Briefly, 4-μm-thick paraffin-embedded tissue sections were deparaffinized, dehydrated, and incubated with protease and pretreated by the Vysis Paraffin Pretreatment IV & Post-Hybridization Wash Buffer Kit (Abbott Molecular Inc.) according to the manufacturer’s instructions [4, 33]. The probe and slides were codenatured at 80 °C for 5 min and incubated at 37 °C overnight in a humidified chamber. Posthybridization washing was performed following standard procedures, and the nuclei were counterstained with DAPI. The slides were examined using a fluorescence microscope with appropriate excitation and emission filters. At least 100 morphologically intact, nonoverlapping nuclei of tumor cells from either myxoid or lipomatous areas were counted. FISH was considered rearranged if more than 10 % of nuclei showed break apart of the dual-color probe signal for the targeted locus.

### RT-PCR for *FUS-DDIT3*

RNA was extracted from fresh-frozen tumor tissues (cases 1, 2, 6–12, 14–21, 23–25, 27–29) as well as formalin-fixed and paraffin-embedded tumor samples (cases 1, 3–8, 13, 22, 26) by using TRIzol® reagent (Life Technologies Japan, Tokyo, Japan) according to the manufacturer’s recommendations. In seven cases of MLSLC (cases 1, 3–8), the paraffin sections were dissected under a microscope, and the samples were taken separately from myxoid and lipoma-like components, which were simultaneously identified by examining the adjacent serial sections stained with H & E.

RNA was reverse transcribed into complementary DNA (cDNA) by PrimScript® II first-strand cDNA Synthesis Kit (Takara Bio, Tokyo, Japan). The PCR to amplify a cDNA that corresponds to *FUS-DDIT3* fusion gene was performed by using KOD-Plus-Ver. 2 (Toyobo, Tokyo, Japan) with the *FUS* ex5 and *DDIT3* primer set amplifying a 160-bp (*FUS*-*DDIT3* type II) fragment and a 436-bp (*FUS*-*DDIT3* type I) fragment or the *FUS* ex7 and *DDIT3* primer set amplifying a 129-bp (*FUS*-*DDIT3* type I) fragment, according to Powers et al. [34]. PCR products were analyzed by Microchip Electrophoresis System for DNA/RNA Analysis with DNA-1000 Kit (Shimadzu, Tokyo, Japan).

## Results

### Clinical features

Clinical information of 29 patients examined is summarized in Table 1. One of five MLSLCs and four of nine ordinary MLSs with follow-up data showed local recurrences. As for WDLS and DDLS, all six retroperitoneal tumors recurred after surgery, whereas four tumors arising in the extremities exhibited no recurrences after a complete wide excision. A remote metastasis was found in one MLSLC, two ordinary MLSs, and one DDLS. One patient with retroperitoneal DDLS was dead of disease

**Table 1:** Clinicopathologic features of liposarcomas with or without lipoma-like component

Case	Age (year)	Sex	Site	Histologic type	Lipoma-like component (%)	Therapy	Follow-up time (month)	Recurrence	Metastasis	Outcome
1	23	F	rt. lower leg	MLSLC	40	Wide excision	66	No	No	ANED
2	66	M	rt. lower leg	MLSLC	40	Wide excision	25	Yes, 1	Yes, 1	ANED
3	75	M	lt. thigh	MLSLC	50	Excision	NA			LTF
4	35	F	rt. lower leg	MLSLC	30	Excision	NA			LTF
5	74	M	lt. thigh	MLSLC	90	Excision	NA			LTF
6	66	F	lt. thigh	MLSLC	30	Wide excision	27	No	No	ANED
7	66	M	rt. groin	MLSLC	70	Wide excision	23	No	No	ANED
8	51	M	rt. thigh	MLSLC	40	Wide excision	8	No	No	ANED
9	28	M	Mediastinum	MLS	0	Excision	60	Yes, 1	Yes, 1	AWD
10	39	F	lt. poplitea	MLS	0	Wide excision	71	No	No	ANED
11	39	F	lt. thigh	MLS	0	Excision	NA			LTF
12	35	M	lt. thigh	MLS	0	Wide excision	75	Yes, 3	No	ANED
13	52	F	lt. thigh	MLS	0	Wide excision	62	Yes, 1	No	ANED
14	54	M	Retroperitoneum	MLS	0	Wide excision	8	Yes, 2	Yes, 1	AWD
15	44	F	Thoracic wall	MLS	0	Wide excision	NA			LTF
16	48	M	lt. knee	MLS	0	Wide excision	22	No	No	ANED
17	53	F	lt. lower leg	MLS	0	Wide excision	52	No	No	ANED
18	36	M	r. buttock	MLS	0	Wide excision	54	No	No	ANED
19	36	F	rt. ankle	MLS	0	Wide excision	51	No	No	ANED
20	69	M	rt. thigh	WDLS	80	Wide excision	49	No	No	ANED
21	48	F	Retroperitoneum	WDLS	70	Excision	69	Yes, 3	No	AWD
22	67	F	Retroperitoneum	WDLS	95	Excision	NA	Yes, 1		LTF
23	74	F	lt. thigh	WDLS	98	Wide excision	23	No	No	ANED
24	50	F	Retroperitoneum	DDLS	40	Excision	104	Yes, 3	Yes	AWD
25	61	M	Retroperitoneum	DDLS	20	Excision	7	Yes, 1	No	AWD
26	73	F	Retroperitoneum	DDLS	70	Incomplete Excision	138	Yes, 1	No	DOD
27	58	F	Retroperitoneum	DDLS	10	Excision	25	Yes, 1	No	ANED
28	83	F	rt. thigh	DDLS	5	Wide excision	33	No	No	ANED
29	79	F	lt. lower leg	DDLS	0	Wide excision	9	No	No	ANED

*ANED* alive and no evidence of disease, *AWD* alive with disease, *DDLS* dedifferentiated liposarcoma, *DOD* dead of disease, *LTF* lost to follow-up, *MLS* myxoid liposarcoma, *MLSLC* MLS with lipoma-like component, *WDLS* well-differentiated liposarcoma

### Macroscopic features

Grossly, MLSs were multinodular or lobulated and often involved the skeletal muscle. The size of tumor was described in six cases of MLSLC, ranging from 7 to 13 cm in the greatest dimension (mean, 9.5 cm; median, 9.5 cm). All eight tumors of MLSLC contained lipoma-like yellowish areas in varying proportions (30–90 %), admixed with myxoid areas resembling ordinary MLS. Foci of necrosis and hemorrhage were found in the large tumors (cases 5 and 8). The myxoid areas of MLSLC and ordinary MLS were typically gelatinous, jelly-like with an abundant myxoid substance.

WDLSs were characterized by large yellowish masses lobulated by fibrous septa. Foci of sclerosis and myxoid changes were often observed. DDLS generally exhibited yellowish lipoma-like components, coexisting with solid gray-whitish areas corresponding to the dedifferentiation. Proportion of lipoma-like to dedifferentiated components varied from tumor to tumor, and one DDLS was composed almost completely of a dedifferentiated component (case 29).

### Histologic features

**Fig. 1:**
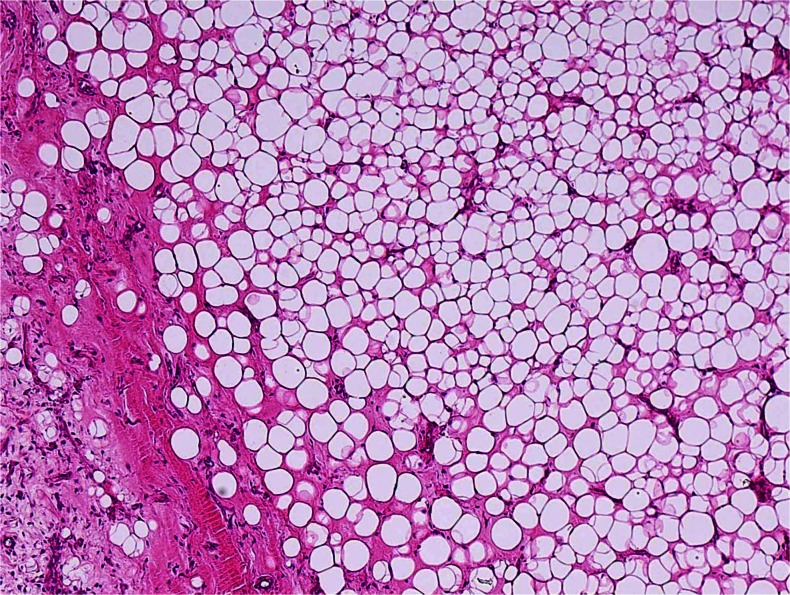
Myxoid liposarcoma with extensive lipoma-like changes. The bulk of this tumor is composed of mature adipocytes with considerable variation in cellular size and shape, bearing superficial resemblance to atypical lipomatous tumor/well-differentiated liposarcoma. Notice a small amount of myxoid component with arborizing capillary blood vessels on the *lower left corner*

**Fig. 2:**
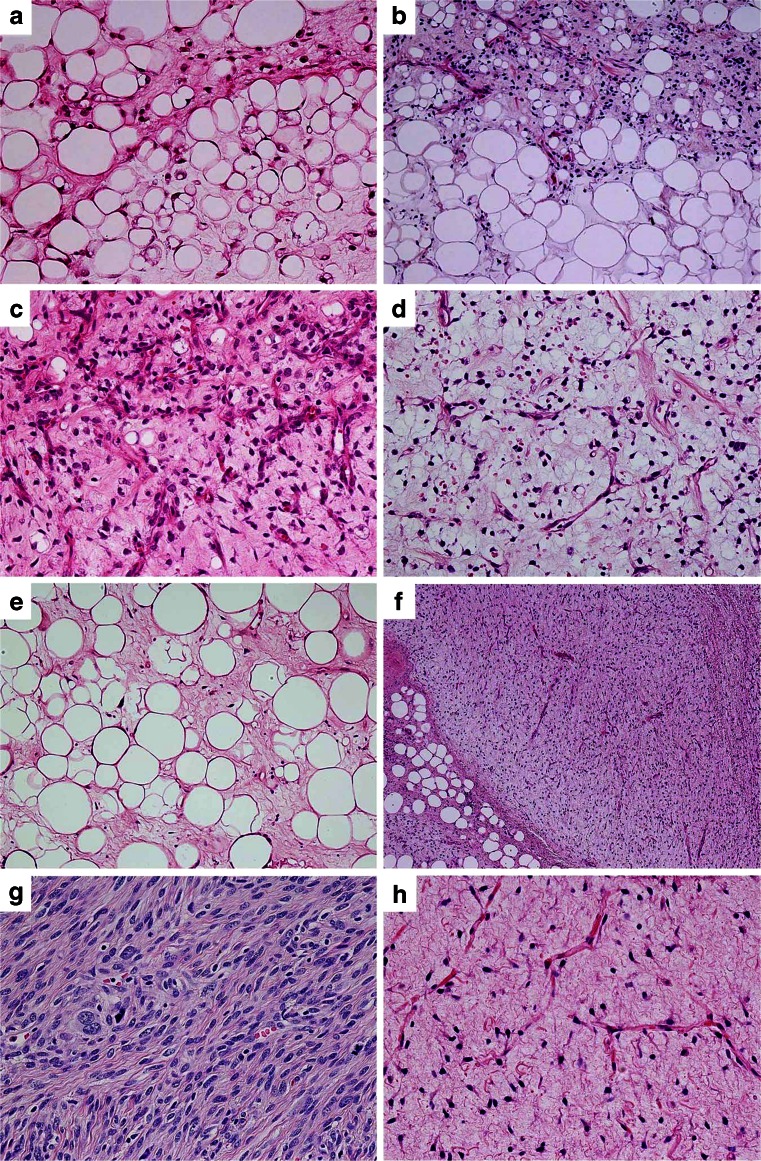
**a**–**c** Myxoid liposarcoma (MLS) with lipoma-like changes (MLSLC). **a** Focal myxoid change in lipoma-like component closely resembling well-differentiated liposarcoma. **b** Transitional zone between myxoid and lipoma-like areas. **c** Myxoid area of MLSLC with immature round cells and signet ring lipoblasts in an abundant myxoid stroma. **d** Ordinary MLS with arborizing capillary blood vessels. **e** Well-differentiated liposarcoma with focal myxoid changes showing similarity to MLSLC. **f** Dedifferentiated liposarcoma (DDLS) exhibits nonlipogenic components with myxoid changes. **g** Undifferentiated pleomorphic sarcoma-like area of DDLS. **h** Marked myxoid changes in DDLS with arborizing capillary blood vessels, reminiscent of MLS

All eight tumors of MLSLC contained lipoma-like well-differentiated component comprising 30–90 % (mean 49 %) of the mass (Table 1, Figs. 1 and 2a, b), whereas the remaining 11 control cases of ordinary MLS lacked distinct lipoma-like components. The lipoma-like components of MLSLC showed abundant mature adipocytes with considerable variation in cellular size (Figs. 1 and 2a), bearing superficial resemblance to WDLS (Fig. 2e). In most tumors of MLSLC, there were gradual transitional zones (Fig. 2b) between the myxoid and lipoma-like components, but some tumor demonstrated focally sharp borders (Fig. 1) between the two components (cases 5 and 8). However, no significant difference of histologic features was found between the myxoid areas of MSLLC and ordinary MLS (Fig. 2c, d). The myxoid areas were made up of a proliferation of small round or stellate cells loosely floating in an abundant myxoid stroma, rich in delicate arborizing capillary blood vessels. Varying numbers of lipoblasts including univacuolated signet ring cells and some small multivacuolated cells were admixed with immature round or stellate cells, but typical spiderweb or giant lipoblasts with bizarre hyperchromatic nuclei were absent.

In all four cases of WDLS, tumors were composed of predominant mature adipocytes interspersed with varying number of atypical cells having hyperchromatic nuclei (Fig. 2e). Multivacuolated lipoblasts with spiderweb features were observed among the tumor cells. Ill-defined focal myxoid areas with spindle or stellate cells were commonly seen in WDLS. DDLS (six cases) contained well-defined nonlipogenic dedifferentiated areas (Fig. 2f–h) in addition to the lipoma-like well-differentiated areas. Both components were usually seen in the same DDLS, but one tumor was composed almost completely of a dedifferentiated component (case 29). The dedifferentiated areas consisted mainly of a high-grade pleomorphic/spindle cell component reminiscent of malignant fibrous histiocytoma (Fig. 2g), but varying degrees of myxoid changes (Fig. 2f, h) were observed in all cases of DDLS examined. In case 24, recurrent DDLS showed prominent cartilaginous metaplasia as well as a low-grade fibromatosis-like component.

### Immunohistochemical findings

**Fig. 3:**
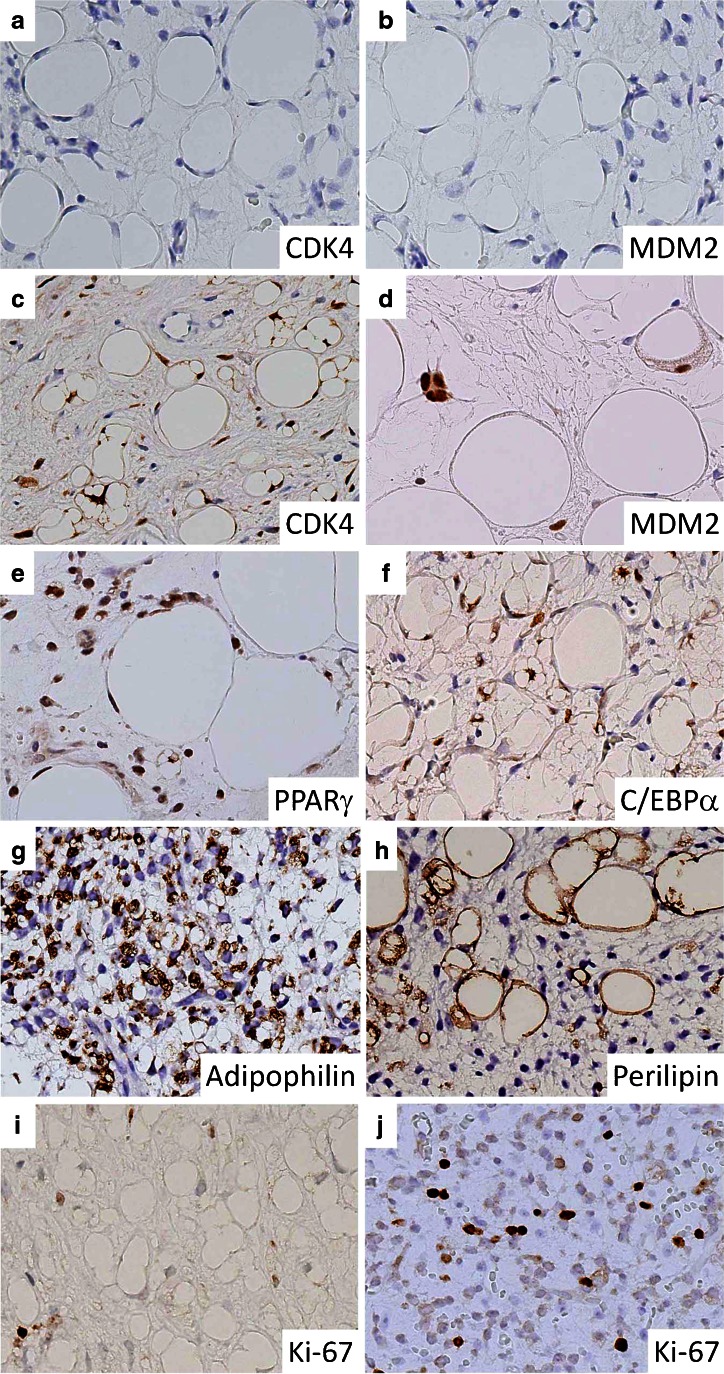
Immunostaining. **a**, **b** Myxoid liposarcoma with lipoma-like change (MLSLC) shows negative staining for CDK4 and MDM2. **c**, **d** Well-differentiated liposarcoma exhibit nuclear expressions of CDK4 and MDM2. **e**, **f** MLSLC shows positive nuclear staining for PPARγ and C/EBPα. **g** Adipophilin is strongly positive in immature lipoblastic cells of MLSLC. **h** Perilipin is positive in large fat vacuoles of mature adipocytes and multivacuolated lipoblasts of MLSLC. **i**, **j** The Ki-67 index is very low in **i** the lipoma-like area of MLSLC, when compared with **j** the myxoid area of the same tumor

In MLSLC, the tumor cells were uniformly negative for CDK4 (Fig. 3a) and MDM2 (Fig. 3b) in both lipoma-like and myxoid areas. Ordinary MLS showed essentially the same immunoreactivity as that of MLSLC. On the other hand, WDLS (Fig. 3c, d) and DDLS were variably positive for CDK4 and MDM2, presenting a sharp contrast to MLS with or without lipoma-like components.

PPARγ and C/EBPα were positive in all tumors of each LS type (Table 2, Fig. 3e, f), but the cells expressing these proteins were somewhat more common in undifferentiated myxoid areas than in lipoma-like components. Adipophilin was expressed in each tumor of MLSLC (Fig. 3g) and ordinary MLS, although myxoid areas contained more positive cells than lipoma-like components did. Generally, reactivity for adipophilin was stronger in immature lipoblastic cells than in mature fat cells. WDLS and DDLS also showed varying positivity for adipophilin in tumor cells, but the numbers of positive cells were usually fewer, when compared with MLS with or without lipoma-like components. Perilipin was positive in all cases of MLSLC (Fig. 3h) and ordinary MLS. However, because the perilipin decollated large fat vacuoles in the cytoplasm, mature fat cells and signet ring lipoblasts as well as multivacuolated lipoblasts revealed a stronger reactivity, when compared with immature cells. In WDLS and DDLS, mature fat cells and spiderweb lipoblasts showed more intense reactivity for perilipin than undifferentiated round or spindle cells did.

**Table 2:** Molecular cytogenetic and immunohistochemical profile of liposarcomas with or without lipoma-like component

Case	Histologic type	Fusion gene (RT-PCR)	*DDIT3* break apart (FISH)	Karyotype	MDM2	CDK4	PPARγ	C/EBPα	Adipophilin	Perilipin	Ki-67 index
			LC/MC		LC/MC	LC/MC	LC/MC	LC/MC	LC/MC	LC/MC	LC/MC
1	MLSLC	*FUS-DDIT3* (type II)	p/p	ND	0/0	0/0	2+/3+	2+/3+	2+/2+	3+/2+	3.0/10.0
2	MLSLC	*FUS-DDIT3* (type I)	p/p	t(12;16), del(6), +8, −15, +mar	0/0	0/0	3+/3+	3+/3+	1+/3+	3+/3+	3.4/11.8
3	MLSLC	*FUS-DDIT3* (type II)	p/p	ND	0/0	0/0	1+/3+	1+/1+	1+/3+	3+/1+	2.2/17.0
4	MLSLC	*FUS-DDIT3* (type II)	p/p	ND	0/0	0/0	2+/3+	2+/3+	1+/3+	3+/3+	3.6/10.6
5	MLSLC	*FUS-DDIT3* (type II)	p/p	ND	0/0	0/0	2+/3+	2+/2+	1+/3+	3+/1+	0.8/7.4
6	MLSLC	*FUS-DDIT3* (type II)	p/p	t(12;16)	0/0	0/0	3+/3+	2+/3+	2+/3+	3+/1+	2.4/12.2
7	MLSLC	*FUS-DDIT3* (type I)	p/p	ND	0/0	0/0	2+/3+	3+/3+	1+/2+	3+/1+	3.0/8.6
8	MLSLC	*FUS-DDIT3*(type II)	p/p	t(12;16)	0/0	0/0	1+/3+	2+/3+	3+/3+	3+/1+	2.8/20.2
9	MLS	*FUS-DDIT3*(type II)	ND	t(12;16), t(1;15)	NA/0	NA/0	NA/3+	NA/2+	NA/3+	NA/2+	NA/22.2
10	MLS	*FUS-DDIT3*(type II)	ND	ND	NA/0	NA/0	NA/3+	NA/2+	NA/3+	NA/1+	NA/8.2
11	MLS	*FUS-DDIT3*(type II)	ND	t(12;16)	NA/0	NA/0	NA/3+	NA/2+	NA/3+	NA/3+	NA/10.2
12	MLS	*FUS-DDIT3*(type II)	NA/p	t(12;16), +8	NA/0	NA/0	NA/3+	NA/2+	NA/3+	NA/2+	NA/6.4
13	MLS	*FUS-DDIT3*(type II)	NA/p	t(12;16), t(1;8), add(9), +mar	NA/0	NA/0	NA/3+	NA/3+	NA/3+	NA/3+	NA/12.8
14	MLS	*FUS-DDIT3* (type I)	ND	t(12;16), +8, +9, +16, +add(16)t(12;16)	NA/0	NA/0	NA/3+	NA/1+	NA/3+	NA/1+	NA/11.4
15	MLS	*FUS-DDIT3*(type II)	ND	t(12;16), +mar	NA/0	NA/0	NA/3+	NA/3+	NA/3+	NA/2+	NA/17.6
16	MLS	*FUS-DDIT3* (type I)	ND	t(12;16), add(6), −22	NA/0	NA/0	NA/3+	NA/2+	NA/2+	NA/1+	NA/17.4
17	MLS	*FUS-DDIT3* (type I)	NA/p	ND	NA/0	NA/0	NA/3+	NA/2+	NA/3+	NA/1+	NA/18.4
18	MLS	*FUS-DDIT3*(type II)	NA/p	ND	NA/0	NA/0	NA/3+	NA/3+	NA/3+	NA/1+	NA/15.6
19	MLS	*FUS-DDIT3*(type II)	ND	t(12;16), +8	NA/0	NA/0	NA/3+	NA/3+	NA/3+	NA/1+	NA/18.4
20	WDLS	n	Uncountable	+1 ~ 3 ring	1+/2+	2+/3+	1+/2+	1+/2+	1+/1+	3+/1+	1.8/4.8
21	WDLS	n	n/n	+1 ~ 2 ring, +mar	1+/2+	3+/3+	3+/3+	2+/2+	1+/1+	3+/1+	4.8/16.0
22	WDLS	ND	n/n	ND	2+/3+	3+/2+	3+/2+	1+/1+	1+/1+	3+/0	2.8/5.8
23	WDLS	n	Uncountable	+Ring	1+/1+	2+/1+	2+/1+	2+/1+	1+/1+	3+/0	1.8/3.4
24	DDLS	ND	Uncountable	+Giant, +ring	2+/2+	2+/3+	1+/2+	1+/2+	1+/1+	3+/0	1.6/10.4
25	DDLS	n	Uncountable	108–115, complex	1+/3+	2+/3+	1+/2+	ND	1+/2+	3+/0	2.8/32.4
26	DDLS	ND	ND	ND	0/2+	2+/3+	2+/3+	2+/2+	1+/1+	3+/1+	0.8/5.2
27	DDLS	ND	n/n	ND	1+/3+	1+/3+	1+/3+	1+/2+	1+/1+	3+/0	5.6/23.0
28	DDLS	n	Uncountable	ND	1+/2+	1+/3+	2+/3+	1+/2+	1+/3+	2+/0	1.0/24.0
29	DDLS	n	NA/n	ND	NA/2+	NA/3+	NA/2+	NA/2+	NA/0	NA/0	NA/35.0

*DDLS* dedifferentiated liposarcoma, *Ki-67 index* percentage of positive nuclei/500 cells, *LC* lipoma-like component, *MC* myxoid component, *n* negative, *MLS* myxoid liposarcoma, *MLSLC* MLS with lipoma-like component, *NA* not available, *ND* not done, *p* positive, *WDLS* well-differentiated liposarcoma; −, 0 %; 1+, 1–25 %; 2+, 26–50 %; 3+, >50 %

The Ki-67 labeling index (Table 2, Fig. 3i, j) was significantly lower in the lipoma-like components of MLSLC (range, 0.8–3.6; mean, 2.7; median, 2.9) than either in the myxoid components of MLSLC (range, 7.4–20.2; mean, 12.2; median, 11.2) (*p* < 0.001) or in the ordinary MLS (range, 6.4–22.2; mean, 14.4; median, 15.6) (*p* < 0.001). In addition, there was no significant difference of the Ki-67 index between ordinary MLS and myxoid component of MLSLC.

### Chromosome analysis

**Fig. 4:**
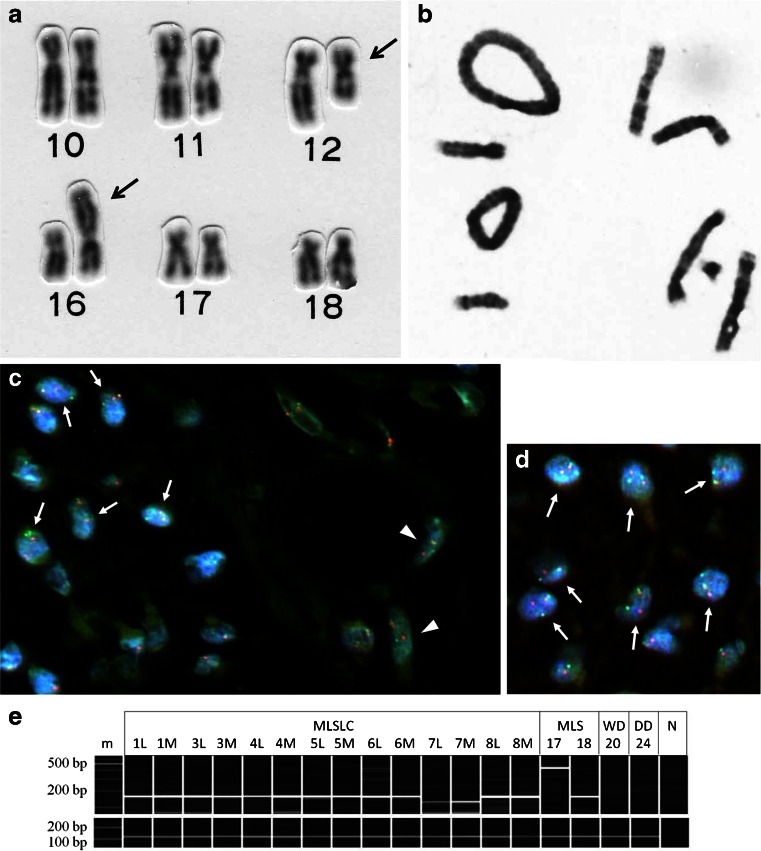
Cytogenetic and molecular analysis of liposarcoma. **a** Partial karyotype of myxoid liposarcoma with lipoma-like changes (MLSLC) (case 2) showing t(12;16)(q13;p11) (*arrows* indicate derivative chromosomes resulting from the reciprocal translocation). **b** Well-differentiated liposarcoma (case 21) exhibits characteristic supernumerary ring chromosomes. **c** Fluorescence in situ hybridization (FISH) detecting *DDIT3* break apart in MLSLC (case 7). In a transitional zone between myxoid (*left*) and lipoma-like (*right*) areas, both immature round cells (*arrows*) and well-differentiated adipocytic cells (*arrowheads*) exhibit break-apart signals of *DDIT3.* **d** Myxoid area of MLSLC (case 8) presenting break-apart signals of *DDIT3* in many sarcoma cells. **e** Detection of *FUS*-*DDIT3* fusion transcript by RT-PCR with the *FUS* ex5 and *DDIT3* primer set amplifying a 159-bp (*FUS*-*DDIT3* type II) fragment (cases 1 to 10, 13, 14, and 16) or a 435-bp (*FUS*-*DDIT3* type I) fragment (case 17) and the *FUS* ex7 and *DDIT3* primer set amplifying a 128-bp (*FUS*-*DDIT3* type I) fragment (case 7). In each MLSLC, *FUS-DDIT3* fusion transcript was found in both lipoma-like (*L*) and myxoid (*M*) areas. Ordinary MLSs (cases 17 and 18) were also positive for the specific *FUS-DDIT3*, whereas well-differentiated (*WD*) and dedifferentiated (*DD*) liposarcomas (cases 20 and 24) showed no fusion transcript. *m*, size marker, 100-bp DNA ladder; *N*, negative control. In the *lower lanes*, the concomitant detection of β_2_-microglobulin gene transcript (120 bp) confirmed the presence of amplifiable RNA

Chromosomal analysis with G-band technique was performed in three cases of MLSLC and in eight cases of ordinary MLS. All MLSLC tumors examined demonstrated t(12;16)(q13;p11) characteristic of MLS (Table 2, Fig. 4a), although a few additional aberrations were found in some cases. Supernumerary ring chromosomes or giant marker chromosomes were never found in MLS with or without lipoma-like components. On the contrary, WDLS (three cases) and DDLS (one case) demonstrated supernumerary ring (Fig. 4b) and/or giant marker chromosomes. In addition, a complex karyotype was found in a case of DDLS.

### FISH for detecting break apart of *DDIT3*

FISH was performed in all eight cases of MLSLC and in four cases of ordinary MLS. Break apart of *DDIT3* was confirmed in each MLS regardless of lipoma-like changes (Table 2). In MLSLC, both lipoma-like and myxoid areas of the same histologic sections showed identical break-apart signals (Fig. 4c, d). The sarcoma cells with *DDIT3* break apart exhibited one yellow (fusion signal), one green, and one red (break-apart signal pattern), whereas normal cells (inner control such as vascular endothelia cells) possessed a two-fusion signal pattern (yellow or red and green overlapping) representing two intact copies of the *DDIT3* gene. On the other hand, WDLS (four cases) and DDLS (six cases) lacked break-apart signals of *DDIT3*. In three cases of DDLS, the break-apart signals were uncountable because of the presence of densely packed multiple signals.

### RT-PCR for *FUS-DDIT3*

*FUS-DDIT3* fusion transcript was found in all tumors of MLS examined, including 8 cases of MLSLC and 11 cases of ordinary MLS (Table 2). The incidence of *FUS-DDIT3* fusion variants was almost the same in MLSLC [type I, two cases (25 %); type II, six cases (75 %)] and ordinary MLS [type I, three cases (27 %); type II, eight cases (73 %)]. In addition, both myxoid and lipoma-like components dissected from the same paraffin sections of MLSLC exhibited the identical fusion variant of *FUS-DDIT3* (Fig. 4e). On the contrary, none of the WDLS/DDLS showed *FUS-DDIT3* fusion.

## Discussion

LSs rarely exhibit unusual histologic features with combined patterns of lipoma-like WDLS and MLS [4, 6, 12, 23]. However, the distinction between MLSLC and WDLS/DDLS with myxoid changes is often difficult because of the morphologic similarities, although the absence of hyperchromatic cells in the adipocytic areas of MLSLC may be a useful diagnostic clue [7]. The recent advances of molecular cytogenetic techniques including FISH and RT-PCR provide powerful tools for resolving these problems, as shown in the present study.

Our study demonstrated that MLSLCs containing peculiar lipoma-like components actually represent MLS, which is different from either true mixed-type LS or WDLS/DDLS with myxoid changes, on the basis of molecular cytogenetic features. Both lipoma-like and myxoid components of the same MLSLC exhibited the identical abnormality, *FUS-DDIT3* specific for MLS, which was confirmed by FISH and RT-PCR. There was a clear cut difference between MLSLC and WDLS/DDLS with myxoid changes; the former was positive for t(12;16)/*FUS-DDIT3* and negative for giant marker/ring chromosomes, whereas the latter exhibited converse characteristics.

The occurrence of true mixed-type LS may be very rare, if it can exist as a distinct entity. Meis-Kindblom et al. [17] described a series of 30 cases of LS including a case of mixed LS, which is composed of WDLS and MLS with a coexistence of ring/giant marker chromosome (characteristic of WDLS) and t(12;16)/*FUS-DDIT3* (specific for MLS). Unfortunately, however, no FISH data from the WDLS component of the tumor was shown in this case. Mentzel et al. [27] reported another example of mixed-type LS consisting of ALT/WDLS and MLS components. By FISH analysis, they found amplifications of *MDM2* and *CDK4* genes in ALT/WDLS areas and translocations of *DDIT3* and *FUS* genes in MLS areas, although their immunohistochemical study failed to demonstrate clear nuclear expressions of MDM2 and CDK4 in the tumor. On the other hand, Antonescu et al. [16] suggested that in many instances of mixed MLS+WDLS or translocation-negative MLS, the tumors may have represented predominantly myxoid WDLS or pleomorphic LS with myxoid changes, as supported by the cytogenetic data in the respective reports. They considered that the presence of microscopic foci of lipoma-like or sclerosing areas, characteristic of WDLS, constitutes sufficient histologic evidence to exclude the diagnosis of MLS, as supported by the consistent absence of *DDIT3* or *FUS* genomic rearrangements in such tumors.

Recently, de Vreeze et al. [5] analyzed eight cases of LS designated as mixed-type LS with combined patterns of WDLS and MLS. By immunohistochemical and molecular data, they concluded that these mixed-type LSs should not be regarded as collision tumors, but as an extreme variant of morphological entity, explaining biological contradiction of mixed-type LS.

In the present study, *FUS-DDIT3* fusion gene was found in distinct lipoma-like components as well as myxoid areas of MLSLC, suggesting that tumor cells of some true MLSs have a potential to differentiate into mature adipocytes producing a lipoma-like masses mimicking WDLS. Conversely, ring/giant marker chromosomes as well as immunohistochemical expressions of CDK4 and MDM2 characteristic of ALT/WDLS were never found in any case of MLS with a lipoma-like component.

Little is known about the mechanism of adipocytic differentiation in MLSLC and ordinary MLS, although various factors have been supposed to be concerned with the development of fat cells. Some molecular studies [13, 35] suggested that *FUS-DDIT3* prevents the development of adipocytic precursors in MLS by repressing PPARγ and C/EBPα. On the other hand, based on a comparative ultrastructural and RT-PCR analysis of MLS/RCLS, Huang and Antonescu [36] found that the variation of *FUS-DDIT3* fusion transcript showed no apparent impact on adipogenesis of MLS, and they considered that other factors might be implicated in their level of differentiation.

Normal adipogenesis is thought to occur in two stages: commitment of mesenchymal stem cells to a preadipocyte fate, followed by terminal differentiation to mature adipocytes [29]. Adipogenic stimuli induce terminal differentiation in committed preadipocytes through the epigenomic activation of PPARγ. The coordination of PPARγ with C/EBP transcription factors maintains adipocyte gene expression.

In our study, both PPARγ and C/EBPα were found in each type of LSs with or without mature lipoma-like components. The results are in accord with the molecular analysis by Tontonoz et al. [14]. Recent studies suggested that PPARγ may regulate the expression of lipid droplet-associated proteins including adipophilin and perilipin. Adipophilin associates with smaller neutral lipid storage droplets located within many tissues, whereas perilipin is located on the surface of larger triacylglycerol droplets in mature adipocytes and on cholesterol ester droplets in steroidogenic cells [30, 31]. In the present study, we found that adipophilin was strongly expressed in tiny fat droplets of immature lipoblastic cells of MLSLC and ordinary MLS, whereas mature adipocytes in lipoma-like component of MLSLC exhibited weak expression of this protein. On the other hand, perilipin showed a strong positive staining in large fat vacuoles of signet ring and multivacuolated lipoblasts as well as mature adipocytes in lipoma-like components of MLSLC and WDLS, but myxoid areas of MLSLC and DDLS contained a few cells possessing small fat vacuoles positive for perilipin. The combined immunohistochemical detection of adipophilin and perilipin may provide a useful ancillary tool for identification of lipoblastic cells in soft tissue sarcomas, since the former is localized in less-differentiated lipoblasts, and the latter is confined to more mature lipoblasts and fat cells.

Immunostaining for Ki-67 (MIB-1) demonstrated a lower labeling index of the nuclei in lipoma-like components of MLSLC, when compared with myxoid areas of the same tumor as well as ordinary MLS (*p* < 0.001). The results suggest that well-differentiated tumor cells resembling mature adipocytes in the lipoma-like components possess a lower level of proliferative activity, whereas undifferentiated or poorly differentiated cells in the myxoid areas of MLS with or without lipoma-like components retain high proliferative activities.

The clinical behavior of LS is highly dependent on the histologic subtype and the location of tumor [1, 2, 4, 17, 25]. The prognosis of MLS involving the deep soft tissue of the extremities is generally favorable when appropriately treated by a complete wide surgical excision with or without a combined radiation or chemotherapy. In the present study, one (20 %) of five MLSLCs followed up had a recurrent tumor but no metastases, whereas four (44 %) of nine ordinary MLSs showed local recurrences and two (22 %) of these patients suffered from remote metastases. It cannot be denied that MLSLC may be less aggressive in clinical behavior when compared with ordinary MLS, although no appropriate statistical analysis could be applied in the present study because of the insufficient numbers of the patients. A large-scale study with sufficient numbers of patients is required to clarify the biological behavior of MLSLC. On the other hand, the overall poor prognosis of retroperitoneal WDLS may result from the frequent occurrence of dedifferentiation, in addition to late tumor detection, involvement of vital structures, and inability to achieve complete resection [1, 2, 25].

In conclusion, our molecular cytogenetic study enabled the distinction between MLSLC and WDLS/DDLS with myxoid changes. The recognition of these peculiar conditions is important, because there are considerable differences of clinical behavior and prognosis between these distinct sarcomas presenting similar morphologic features. In addition, the combined immunohistochemical detection of adipophilin and perilipin may provide a useful ancillary tool for identification of lipoblastic cells in soft tissue sarcomas, since the former is localized in less-differentiated lipoblasts, and the latter is chiefly confined to more mature lipoblasts and fat cells.
